# In Vitro Propagation of XXY Undifferentiated Mouse Spermatogonia: Model for Fertility Preservation in Klinefelter Syndrome Patients

**DOI:** 10.3390/ijms23010173

**Published:** 2021-12-24

**Authors:** Guillermo Galdon, Nicholas A. Deebel, Nima Pourhabibi Zarandi, Mark J. Pettenati, Stanley Kogan, Christina Wang, Ronald S. Swerdloff, Anthony Atala, Yanhe Lue, Hooman Sadri-Ardekani

**Affiliations:** 1 Wake Forest Institute for Regenerative Medicine (WFIRM), Wake Forest School of Medicine, Winston-Salem, NC 27101, USA; galdon.guillermo@gmail.com (G.G.); ndeebel@wakehealth.edu (N.A.D.); npourhab@wakehealth.edu (N.P.Z.); skogan@wakehealth.edu (S.K.); aatala@wakehealth.edu (A.A.); 2 Facultad de Medicina Universidad de Barcelona, 08036 Barcelona, Spain; 3 Department of Urology, Wake Forest School of Medicine, Winston-Salem, NC 27101, USA; 4 Section of Medical Genetics, Department of Pathology, Wake Forest School of Medicine, Winston-Salem, NC 27101, USA; pettenat@wakehealth.edu; 5 Division of Endocrinology, The Lundquist Institute and Harbor-UCLA Medical Center, Torrance, CA 90502, USA; wang@lundquist.org (C.W.); swerdloff@lundquist.org (R.S.S.); Lue@lundquist.org (Y.L.)

**Keywords:** spermatogonia, spermatogonia stem cells, Klinefelter syndrome, male infertility, fertility preservation

## Abstract

Klinefelter syndrome (KS) is characterized by a masculine phenotype, supernumerary sex chromosomes (usually XXY), and spermatogonial stem cell (SSC) loss in their early life. Affecting 1 out of every 650 males born, KS is the most common genetic cause of male infertility, and new fertility preservation strategies are critically important for these patients. In this study, testes from 41, XXY prepubertal (3-day-old) mice were frozen-thawed. Isolated testicular cells were cultured and characterized by qPCR, digital PCR, and flow cytometry analyses. We demonstrated that SSCs survived and were able to be propagated with testicular somatic cells in culture for up to 120 days. DNA fluorescent in situ hybridization (FISH) showed the presence of XXY spermatogonia at the beginning of the culture and a variety of propagated XY, XX, and XXY spermatogonia at the end of the culture. These data provide the first evidence that an extra sex chromosome was lost during innate SSC culture, a crucial finding in treating KS patients for preserving and propagating SSCs for future sperm production, either in vitro or in vivo. This in vitro propagation system can be translated to clinical fertility preservation for KS patients.

## 1. Introduction

Klinefelter syndrome is a chromosomal alteration presenting with a masculine phenotype, with one Y chromosome and at least two X chromosomes (47 XXY being the most common genotype) [1]. It affects around 1 out of 650 newborn males and, despite remaining widely underdiagnosed, represents the leading genetic cause for male infertility [2,3]. The most common pathologic finding in Klinefelter adult patients is small testis due to seminiferous tubule fibrosis [4]. However, little is known about the preceding pathophysiologic process except that germ cell loss starts in the fetus [5,6,7,8], accelerates around the onset of puberty [9], and progresses to azoospermia in most adult patients [10,11].

Several authors have studied this process of progressive germ cell loss [9,10,12], resulting often in hyalinized or Sertoli cell only seminiferous tubules. However, it remains unclear if the germinal loss is due to an intrinsic dysfunction of the germ cells [8], a dysfunction of supporting Sertoli cells [13], or defective spermatogonial stem cell (SSC) renewal [14]. Studies analyzing these germ cells entering meiosis have described a meiotic arrest either at spermatogonia [9] or early spermatocytes [15]. In addition to germ cell loss, Leydig cell hyperplasia is characteristic in KS patients [4,9].

Testosterone production and testicular growth are often within the normal range during early puberty, whereas LH, FSH, and estradiol concentrations are increased. Serum testosterone concentration decreases when mid puberty is reached and remains low or in a lower than normal range in adult KS patients [16]

Despite extensive testicular fibrosis and germ cell depletion in the testis, it has been shown that adult KS patients may have focal areas of conserved spermatogenesis in the testis [1,4,9,10,17]. Moreover, few KS patients may still have some spermatozoa present in the ejaculate [1,18] that could be potentially used for in vitro fertilization (IVF) or intracytoplasmic sperm injection (ICSI) [19,20,21].

Current therapeutic options for adult KS patients presenting with non-obstructive azoospermia in the ejaculate include surgical testicular sperm extraction (TESE), with or without utilization of a surgical microscope, followed by ICSI [14,22,23]. According to recent meta-analyses, the success rate of sperm retrieval using TESE in KS patients is up to 47% regardless of the age of the patient at the time of surgery and should be decided on a patient to patient basis [24,25]. Moreover, the subsequent pregnancy rate using harvested sperm is 43% per ICSI cycle [24,26].

There have also been reports of spermatogonia in the testes of patients without intratesticular spermatazoa on micro-TESE [27,28]. These findings suggest that SSC technology could be an alternative option in the future.

It has been hypothesized that novel cell therapies may allow the use of autologous cryopreserved testicular tissue to treat Klinefelter patients’ infertility [29,30,31,32]. However, a significant challenge to prove this concept is the lack of acceptance for performing testicular biopsy before puberty when there are still a significantly higher number of undifferentiated spermatogonia in Klinefelter testes as shown based on either H&E or IHC staining [9,12,33,34]. The effectiveness of these therapies might also be influenced by testosterone replacement treatment, making the timing of the testicular biopsy a vital issue for discussion amongst the patient, their family, and their clinician [33]. More consistent data is needed to establish a better standard of care for KS patients [27,28].

Several Klinefelter animal models have been introduced in the past decade to better understand the disease [35,36,37,38,39,40,41,42,43]. Our group established a colony of the most advanced 41 XXY mouse models sharing many clinical characteristics with human 47XXY [35,36,37]. Previous studies found that gonocyte numbers were similar in XY and XXY mice at postnatal day 1, followed by an over 60% decrease in the number of gonocytes in the XXY mice on day 3 and further progressive loss of spermatogonia by days 5 and 7. Only a few spermatogonia remained in the XXY mice testis on day 10, mimicking the findings in pubertal boys [36].

One of the main limitations of potential SSC-based fertility treatments [44,45] in KS patients is the scarcity of SSC in small testicular biopsies. To make autologous therapies feasible [44,45], it is critically essential to propagate these cells in culture and increase their number successfully. SSCs’ in vitro propagation has been developed in different species in the past two decades, including wild-type mice [46,47,48,49]. We also reported the first SSC in vitro propagation system using adult [50] and prepubertal [51] cryopreserved human testis tissue. This study aimed to culture testicular cells from frozen prepubertal XY (as a control) and XXY mice testes to maintain and increase the population of putative SSCs. This technique may overcome the germ cell loss observed at puberty [36].

## 2. Results

### 2.1. Histology and Immunohistochemistry (IHC) Comparison of XY and XXY Neonatal Mouse Testes

**Figure 1:**
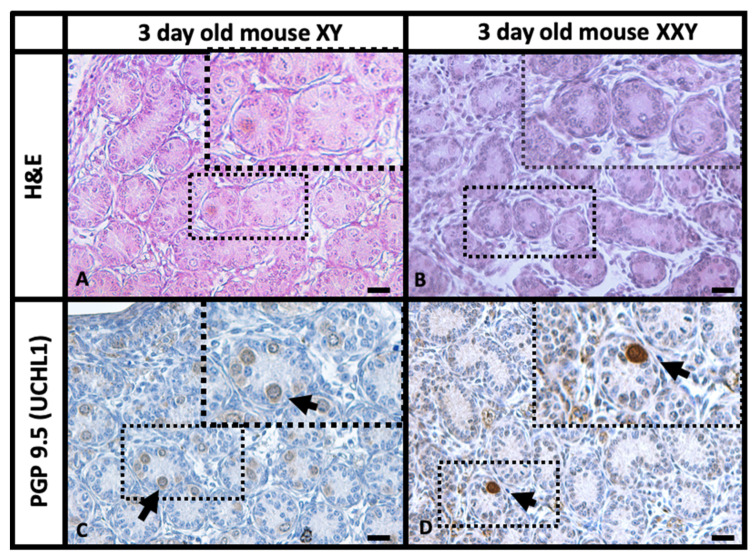
Histology images from a paraffin section of the whole testis tissue with H&E and IHC for PGP9.5 (UCHL1) as an undifferentiated spermatogonia marker. Hematoxylin and Eosin (H&E) staining of 3-day-old XY (**A**) and XXY (**B**) mice testes. UCHL1 staining of 3-day-old XY (**C**) and XXY (**D**) mice testes. Arrows point to cells positive for UCHL1 (DAB, brown color) inside the seminiferous tubules, most likely gonocytes. Scale Bar 20 µm.

Histologic analysis of testes from both XY and XXY mice was performed to assess the effect of the extra X chromosome on 3-day-old animals. Hematoxylin and Eosin (H&E) staining (Figure 1A,B) showed XXY mouse testis had a tubular structure that was primarily conserved. Although, the number of undifferentiated spermatogonia appeared to be lower in the XXY mouse compared to that in XY control mice (Figure 1C,D). Interstitial testicular tissue was more prominent in XXY mouse testes than in XY control mice, with the presence of characteristic Leydig cell hyperplasia as previously described [37].

The immunohistochemistry for PGP 9.5 (UCHL1) results (Figure 1) were especially useful as this marker is expressed in undifferentiated spermatogonia from seminiferous tubules and Leydig cells in the testicular interstitium tissue [52] but not in other somatic cells within the testis. The staining in 3-day-old XY mouse testis showed bright positive signals on intraluminal gonocytes and undifferentiated spermatogonia migrating to the basal membrane as well as a dimmer signal on interstitial cells. When the same staining was performed on 3-day-old XXY mouse testis, very few positive cells were observed inside the seminiferous tubules or migrating to the basal membrane. Nevertheless, interstitial cells showed prominent signals suggesting the presence of Leydig cell hyperplasia and validating the staining. These findings are consistent with previous characterizations of the XXY mice [35,36,37].

### 2.2. Isolation and Culture of XXY Neonatal Mouse Testicular Cells

The testicular cell isolation process was performed based on both mechanical and enzymatic dissociation of testicular tissue as described by Shinohara’s group [46,47,48,49] and our previous works [50,51]. Supplemented StemPro-34 is a widely used media for testicular cell culture and has already shown promising results in both mice [46,47,48,49] and men [50,51]. However, for this experiment, a different enriched version of StemPro media under the name of “Gonomedia” (details in the methods section) was used. Our group proposes that Gonomedia better supports neonatal mice testicular cell expansion.

The frozen testes came from the Klinefelter XXY mice maintained at The Lundquist Institute and Harbor-UCLA Medical Center kept frozen at −80 °C and sent to Wake Forest Institute for Regenerative Medicine (WFIRM) [35,36,37]. The same isolation and culture method was applied to both the 3-day-old XXY mouse and its XY littermate mice in parallel. For each isolation, 5 frozen testes were pooled for a total weight of 1 mg. After mechanical and chemical tissue digestion, 300,000–350,000 cells were retrieved and seeded in Gonomedia.

**Figure 2:**
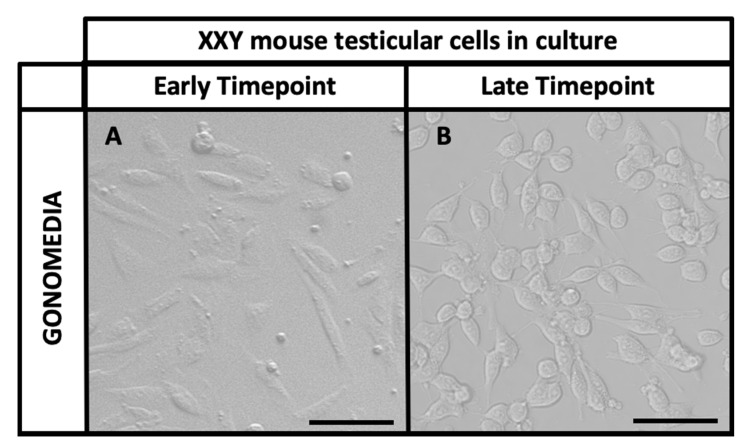
Optic microscopy images of XXY mouse testicular cells in culture: (**A**) Cells re-seeded in Gonomedia after five days in culture (1st passage, Early timepoint). (**B**) Cells re-seeded in Gonomedia after 89 days in culture (10th passage, Late timepoint). Scale Bar 20 µm.

In our isolation and culture system of testicular cells [50,51], both somatic and germ cells coexist, supporting and nurturing the expansion of each other. The former population presents an elongated morphology and usually attaches to the culture dish early, creating intercellular connections and forming patches. The latter population presents a small round form and usually attaches later, using the somatic cell patches as a physiologic feeder layer (Figure 2).

**Figure 3:**
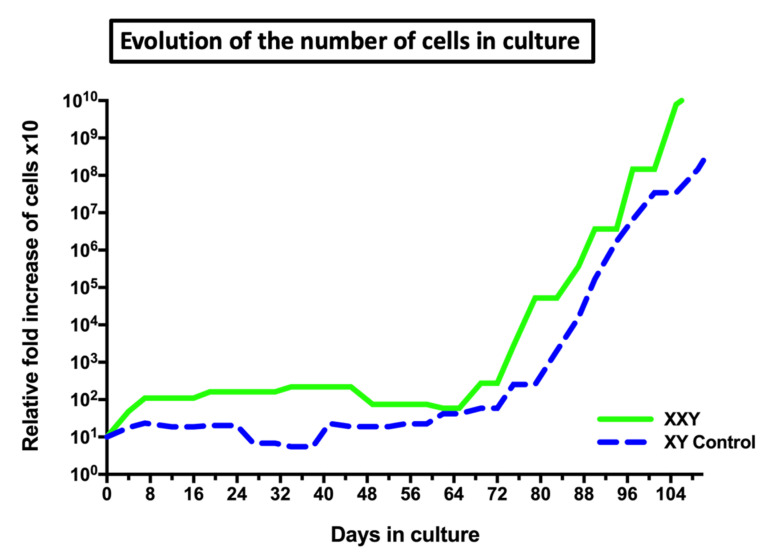
Evolution of the number of cells in culture from XY and XXY mice testes. Both conditions include data from two replicas of cell isolation from 5 testes each (*n* = 10) seeded in Gonomedia to improve the transition from gonocyte to spermatogonia and potentiate cell propagation.

In our laboratory, testicular cells from XXY mice remained viable and stable in culture with a discrete propagation rate, less than a 10-fold increase, during the first 60 days. However, after 65–70 days in culture, cells started to expand exponentially at least until day 110, when the study goal was considered met and the study was terminated. At this point, cells in culture had increased over one million fold its number (Figure 3). To our best knowledge, this is the first report of cells from XXY testes being successfully propagate in long-term cultures, overcoming the previously known period of apoptosis in the XXY mouse model. Testicular cells from XY mice testes, as the control, grew in similar pattern in parallel (Figure 3).

### 2.3. Gene Expression Analyses of Cultured Cells

Different spermatogonia markers were used to assess the character and differentiation of the cells in culture. This was crucial because only undifferentiated spermatogonia cells containing an SSC subpopulation can repopulate seminiferous tubules after transplantation.

**Figure 4:**
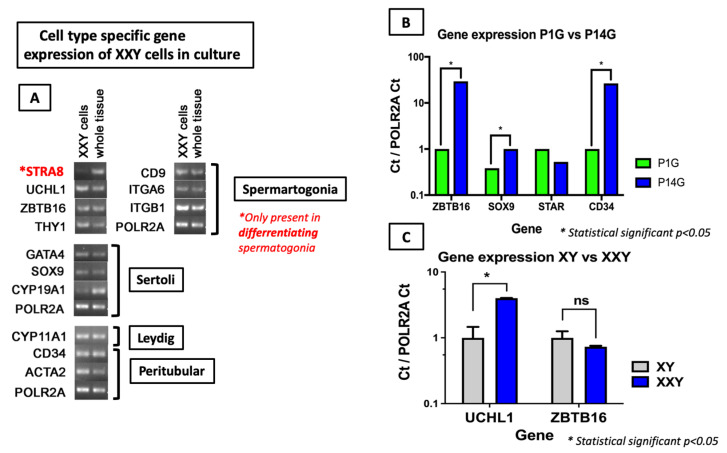
Gene expression analysis of cultured cells from XXY testes. (**A**) qPCR analysis of XXY cells after 31 days in culture showed significant expression of markers from all four major testicular cell types: spermatogonia, Sertoli, Leydig, and peritubular cells. (**B**) Comparative gene expression between XXY testicular cells from early (Passage 1, 7 days in culture) and late time points (Passage 14, 110 days in culture) in Gonomedia using paired t student comparison via Prism9 software; (**C**) Comparative gene expression between XXY and XY testicular cells that were long-termed cultured (Passage 14, 110 days) using a t student comparison via Prism9 software; Results expressed as a Delta Delta Ct value normalized with the housekeeping gene POLR2A. Statistically significant *p* < 0.05 shown as *.

Characterization of cultured XXY cells confirmed positive expression for markers of all four major cell types expected in the immature mouse testis: undifferentiated spermatogonia (THY1, CD9, ITGA6, ITGB1), Sertoli (GATA4, SOX9, CYP19A1), Leydig (CYP11A1), and peritubular (a heterogeneous population with multiple cell types including peritubular myoid cells and telocytes [53]) (CD34, ACTA2) cells (Figure 4A).

To detect differences in gene expression from XXY cells in culture, qPCR was performed at an early timepoint (Passage 1, 7 days in culture) and late timepoint (Passage 14, 110 days in culture) in the same culture media. Results show a significant increase in the increment in gene expression for markers of non-differentiated spermatogonia (ZBTB16), Sertoli cells (SOX9), and peritubular cells (CD34), while no significant difference was found in the Leydig cell (STAR) marker (Figure 4B).

Undifferentiated spermatogonia markers PGP9.5 (UCHL1) and ZBTB16 were highly expressed in cultured cells from both XXY and XY testes at several checkpoints along its time in culture. When expression levels between XXY and XY cells were compared, PGP9.5 (UCHL1) expression was higher in XXY than XY cells while ZBTB16 expression levels were comparable (Figure 4C). The expression of the differentiating spermatogonia marker STRA8 was undetectable in both cultured cells from XY (data not shown) and XXY (Figure 4A) testes, indicating that cells in culture conserve a non-differentiated status potentially suitable for transplantation or use for in vitro differentiation.

**Figure 5:**
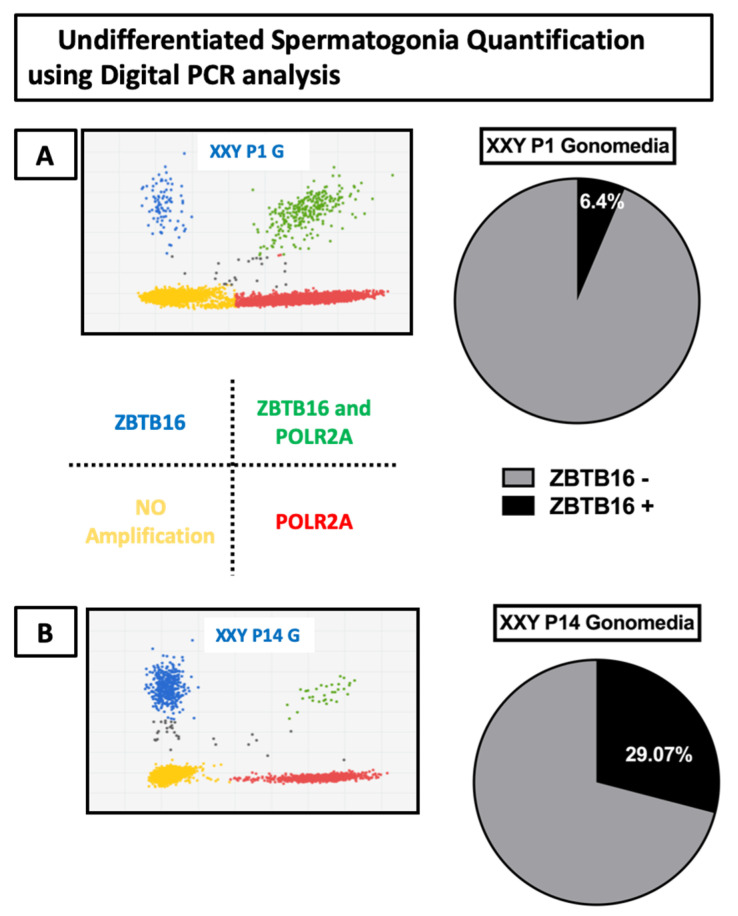
Digital PCR analysis of gene expression for the undifferentiated spermatogonia marker ZBTB 16 (FAM) and housekeeping gene POLR2A (VIC): (**A**) Gene expression from XXY mouse testicular cells after seven days in culture (1st passage) in Gonomedia; (**B**) XXY mouse testicular cells after 110 days in culture (14th passage) in Gonomedia.

In addition to quantitative gene expression analysis, digital PCR was used to assess the portion of all cells expressing the ZBTB16 undifferentiated spermatogonia marker, compared to a POLR2A housekeeping gene. After seven days in culture, the results showed that 6.4% of cells expressed ZBTB16. Then, after 110 days in culture, digital PCR analysis was repeated, showing 29.07% of ZBTB16-positive cells (Figure 5).

### 2.4. Flow Cytometry Analyses of Cultured Cells from XXY Testes

**Figure 6:**
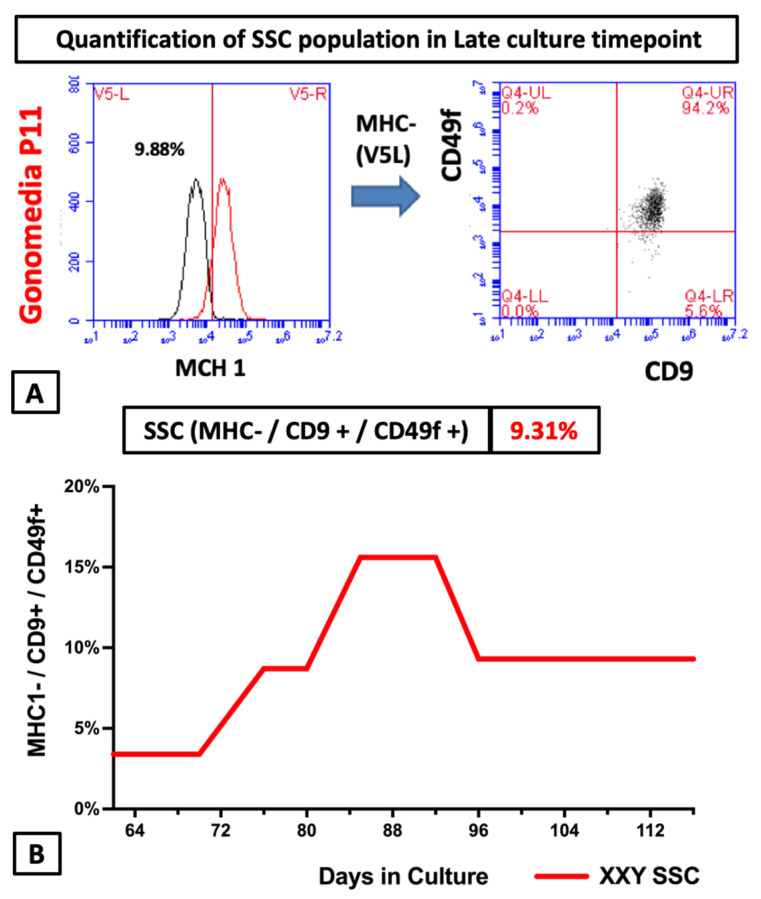
(**A**) Flow cytometry analysis to identify putative spermatogonial stem cells (MHC I- CD9+ CD49f+) XXY mouse testicular cells after 96 days in culture (11th passage) in Gonomedia; (**B**) Evolution of the percentage of putative SSC (MHC I- CD9+ CD49f+) along with culture.

When enough cells were propagated in culture, flow cytometry was used to identify the characteristic protein expression of the enriched cells that are SSC. To date, there is no single marker to identify the SSC population. On the other hand, the combination of MHC I-/CD9+/CD49f+ is well described in the literature as an enriched SSC population and a good predictor for SSC [54,55,56,57,58,59] transplantation success. The same analysis was repeated at several time points during our culture process (Figure 6).

A putative SSC population was identified in all conditions at every checkpoint in the study. The percentage of cells expressing SSC potential markers remained between 3.3 and 18.8% (Figure 6). These values were comparable to analysis of XY mouse controls testicular cells (data not shown). These findings are promising and suggest that in vitro propagated cells using the described culture system could play a role in SSC transplantation.

### 2.5. DNA FISH Analysis for X and Y Chromosomes

**Figure 7:**
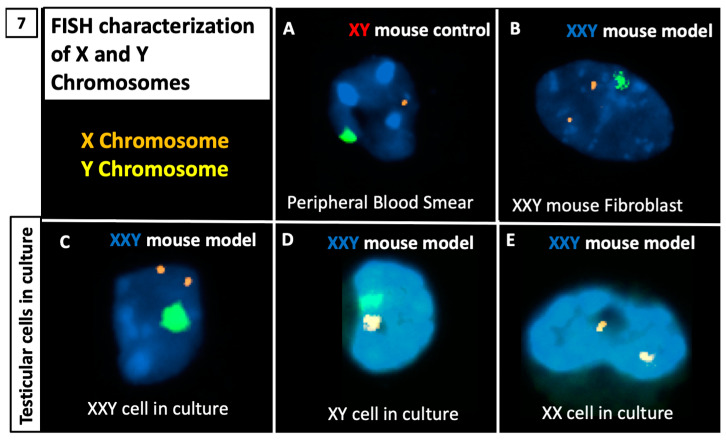
Characterization of Klinefelter (XXY) mouse and isolated testicular cells using FISH: (**A**) FISH staining on peripheral blood smear from control, XY mouse; (**B**) FISH staining on skin fibroblast from the Klinefelter model XXY mouse before sacrifice; (**C**–**E**) FISH staining on isolated and propagated testicular cells from the Klinefelter model (XXY) mouse testes; variations of XXY, XY and XX cells were identified in KS testicular cells in culture.

FISH analysis was performed on the samples to characterize X and Y chromosomes. Cells were cytospinned into slides for chromosome hybridization with fluorescent probes showing specific staining for X (orange) and Y (green) chromosomes. Our system correctly identified both X and Y chromosomes in an XY control mouse blood smear and XXY mouse specimen’s fibroblasts. Then, the same experiment was reproduced with cultured testicular cells from the XXY mouse testes (Figure 7).

**Figure 8:**
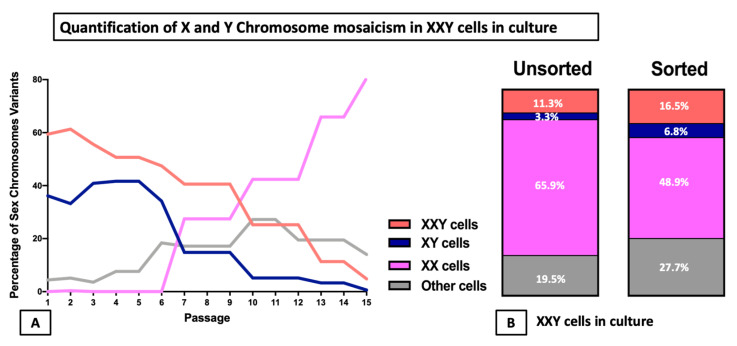
To quantify the presence of X and Y chromosomes in the testicular cell culture from the XXY mice, FISH staining and systematic counting were performed in consecutive passages. (**A**) Evolution of the different mosaic populations in culture. (**B**) Comparison of mosaicism of the cells 96 days in culture (12th passage) between unsorted and FACS sorted for putative SSC (MHC I- CD9+ CD49f+).

Systematic analysis of the X and Y chromosomes from cells in culture at different time points was carried out to identify diverse cell populations and quantify possible mosaicism. From each cyto-spun slide, five representative areas were imagined, and every cell was classified depending on its number of X and Y chromosomes. Both stationary and dividing cells were included. The results showed that mosaicism was present at the initiation of the culture. Initially, the most prevalent population was XXY cells accounting for 60% of the cells in culture, followed by XY cells representing 35%, and the final 5% was a mix of XX, XYY, and XXXY cells. For the first eight passages, the percentages of both XXY and XY remained most common although slowly decreased while XX and XXXY populations became more common. After passage 10, the XX population became the most common, representing at least 42% of the cells in culture while XXY and XY cells followed with 28% and 5%, respectively. From then on, the XX population continued to increase up to 80% of the cells in culture at passage 15 (Figure 8A).

In a subsequent study, we assessed if germ or somatic cells preferentially presented a particular karyotype. Parallel FISH analysis was performed in unsorted and FACS-sorted for MHC-/CD9+/CD49f+, as previously described as an enriched SSC population. The results showed a more significant proportion of XXY cells in sorted cells than unsorted (16% vs. 11%) as well as XY cells (6.7% vs. 3.2%), while XX cells were more frequent in the unsorted samples than the sorted (65% vs. 48%) (Figure 8B).

## 3. Discussion

Previous studies have thoroughly characterized the 41 XXY mice as a model for Klinefelter syndrome and showed that the number of spermatogonia significantly decreased in XXY mice by day three after birth [35,36,37]. Histological analysis carried out in this study showed alteration of the testis architecture with empty seminiferous tubules and prominent interstitial PGP9.5 (UCHL1) positive cells. These findings support that XXY mice are a reliable model for Klinefelter patients presenting early germ cell loss and Leydig cell hyperplasia as is seen in the human KS patient.

The 3-day-old XXY mice were expected to show some degree of germ cell loss without reaching complete depletion. At this age, the mice would mimic a scenario where peripubertal or adult KS patients with testicular fibrosis but not complete germ cell depletion may undergo testicular biopsy [27,32,33,34,60]. With the same clinical setting in mind, a decision was made to used frozen mice testes as this would be translatable to pre-pubertal Klinefelter patients that undergo testicular biopsy and cryopreserve testicular tissue for fertility treatments later in their life [28,29,30,31].

The culture system used in this study was optimized for the neonatal XY mouse testes before attempting to apply it on isolated cells from the XXY mice testes. This step was necessary because of the well-known challenges with growing 3-day-old C57Bl6 mice testicular cells in vitro [49]. One possible explanation is that after birth, the germ cell population of mice is transitioning from quiescent gonocytes [61] into mitotic spermatogonia [48], which may represent a delay for in vitro propagation. Two different culture surfaces were tested: regular plastic culture plates and laminin-coated culture plates. The rationale to test laminin-coated wells as a culture surface follows some reports that suggested supporting cell attachment and proliferation [46]. A decision was made not to use a commercially available cell-based feeder layer that other authors have reported to improve neonatal mouse SSC attachment [48,62]. The explanation is that these biological products are not approved for clinical application, making the culture system not translatable to clinical settings. We also tested two different media: supplemented StemPro, which has been previously shown to support mouse SSC propagation in vitro [46], as well as our formulation called Gonomedia. This is a variant of supplemented StemPro enriched with 20% FBS, Follistatin, PDGF, and FSH (see the method section for more details). Based on the present literature [63,64], our group hypothesized that Gonomedia could help push quiescent neonatal gonocytes into spermatogonia and favor cell propagation. In our hands, the laminin-coated culture surface did not improve cell attachment nor cell propagation.

In our experience, a higher cell seeding concentration potentiates cell attachment but limits the propagation yield of cells in culture. For this study, seeding concentrations from 3500 to 25,000 cells/cm^2^ were tested. In our hands, only Gonomedia combined with the plastic surface and up to 250,000 cells/cm^2^ seeding concentration allowed cells to propagate initially while conserving the gene expression and surface marker characteristics of SSC during the first 55–70 days in culture. Nevertheless, once cells started propagating consistently, we reduced the seeding concentration to as low as 3000 cells/cm^2^ to optimize the yield of cell propagation with no negative impact on cell behavior.

The cells usually took between 55 and 70 days to start growing steadily, with subsequent exponential propagation of both XY and XXY cells. The initial quiescent stage suggested by these findings is similar to what was previously reported by Kanatsu-Shinohara et al. on neonatal mouse testicular cell culture, although our novel formulated Gonomedia was not used previously.

Another promising finding of this study was the presence of all significant testicular cell types (Spermatogonia, Sertoli, Leydig, and peritubular cells) within the culture. Maintaining the viability of all different cell types is paramount to recreating the in vivo physiology of the testes. However, more studies could be done to assess specific up- and downregulated gene expression pathways of every cell type to identify new treatment targets. Moreover, when we compared the ZBTB16 expression level in cultured testicular cells from XXY and XY mice, both presented a similar expression level while PGP9.5 (UCHL1) was significantly higher in XXY cultured cells (Figure 4C). ZBTB16 is highly specific for undifferentiated spermatogonia while PGP9.5 (UCHL1) is expressed in both spermatogonia and Leydig cells [52]. Therefore, the difference in the UCHL1 expression level in XXY culture is most likely related to Leydig cells’ hyperplasia in KS testes. Although a final answer is not provided at this point in our study, these results suggest that some of the differences between in vitro cultured testicular cells from XY and XXY mice are relevant to in vivo environments. Therefore, it can be considered as a tool for future physiologic studies.

Consecutive DNA FISH analysis in cultured cells from KS mouse testes demonstrated sexual chromosome mosaicism from the onset of the culture. The populations of cells in culture varied significantly over time. At first, most cells presented with XXY, although a significant population of XY was present with a scarce population of XX. Later in the culture process, both XXY and XY populations experienced a substantial decline mirrored by a significant increment of the XX population. This suggests instability in the Y and extra X chromosomes in this KS mouse model. A similar phenomenon has been described as trisomy-biased chromosome loss (TCL) in induced pluripotent stem cells (iPSCs) derived from KS mice fibroblasts [38].

In previous studies characterizing XXY mice, karyotype was performed on cultured fibroblasts. However, in this study, we analyzed hundreds of cultured testicular cells using FISH at each timepoint of the culture, detecting the populations that may have remained concealed in the past studies. Another critical point is long-term culture may alter the chromosome stability of cells. However, previous reports have looked deeply into this matter without finding definitive reasons for concern [65,66]. Additionally, the presence of few mosaic testes between the XXY mice pooled before cell isolation may contribute to the mixture population of XY and XXY cells at the beginning of cell culture. Although the percentage of the XY population in propagated KS testicular cells decreased during the culture (Figure 8), the cumulative number of XY cells increased dramatically from around 120,000 (40% of 300,000 cells) to at least 9,000,000,000 (3% of 3 × 10^12^ cells) (Figure 3 and Figure 8). The finding of XX cells in late passage but not before culture demonstrated that the trisomy-biased chromosome loss (TCL) occurred in XXY mouse testicular cell culture [38]. Importantly, these data indicated that tissue- and organ-specific mosaicism may cause a variety of phenotypes in XXY aneuploidy [67].

We have shown previously that testes from adult XXY mice were successfully repopulated after SSC transplantation from XY littermates [36]. In the current study, testicular cells from XXY mice were isolated and propagated in culture and, using flow cytometry, a putative SSC population (MHC I-/CD9+/CD49f+) was identified. Therefore, we believe that a population of SSC may remain viable after initial germ cell loss. This needs to be proven by transplantation or in vitro differentiation of cultured XXY testicular cells to haploid germ cells in the future.

Taking these findings into account, along with previous reports of preserved foci of spermatogenesis in adult KS [1,4,9,10,17], the data suggest that adult human Klinefelter testes could potentially sustain spermatogenesis using SSC technology. A critical step to bring this therapy to the clinic setting would be to expand cultured human KS SSCs to the point that either autologous SSC transplantation or in vitro differentiation could be possible. We believe this study is the first step in that direction. Moreover, as previously mentioned, the specific conditions for this study were selected to support an efficient technique in a clinical setting. Therefore, we hope these data will help to establish an SSC culture system for KS patients.

## 4. Materials and Methods

### 4.1. Animals

In total, 41 XXY mice (*n* = 5) and 40XY littermates (*n* = 5) with a C57Bl6 genetic background were generated at the Lundquist Institute lab as described previously [35,36,37]. Immediately after birth, XXY puppies were identified using DAPI staining on fibroblast spread. The 3-day-old mice were sacrificed, and orchiectomy was performed under a sterile technique. Each testis was cut in half and cryopreserved in 1.5 mL cryovials (VWR, PA, USA) using cryopreservation solution: MEM (Invitrogen, MA, USA), 20% Fetal Bovine Serum (FBS, ThermoFisher Scientific, MA, USA), and 8% dimethyl sulfoxide (DMSO, Mylan, WV, USA). An isopropyl alcohol-based constant slow freezing device (Mr. Frosty, Nalgene, Sigma-Aldrich, MO, USA) was used to freeze the samples −1 °C/min up to −80 °C overnight [50,51,54,66]. The next day, cryovials were transferred into liquid nitrogen tanks for long-term storage. No experiments with living animals were performed. The creation of 3-day-old XXY mice for testicular cell culture was done at THE LUNDQUIST INSTITUTE FOR BIOMEDICAL INNOVATION AT HARBOR-UCLA MEDICAL CENTER under LUNDQUIST INSTITUTE IACUC approval #32014-02 (Ref#052444), last renewal 2/18/2021 through 2/17/2022.

### 4.2. Histology, Immunohistochemistry, and Viability Assay

Freshly recovered testes from XY and XXY mice were fixed in Bouins for 2 h and then transferred to 70% ethanol. The tissue was washed three times for 5 min in 70% ethanol before processing for paraffin embedding. Samples were then paraffinized using an automated system (Leica ASP300S, Buffalo Grove, IL, USA) following increasing concentrations of isopropyl alcohol followed by xylene and then paraffin on a three-hour program. Paraffinized tissue was then embedded into blocks. Using a Leica RM 2255 manual microtome, 5 µm thick sections were obtained and placed on histology glass slides. Slides were then placed in a 60 °C oven for 30 min to eliminate the excess paraffin.

#### 4.2.1. Hematoxylin and Eosin Staining

Hematoxylin and Eosin (H&E) staining was performed using an automated stainer (Leica autostainer XL, Buffalo Grove, IL, USA) following a house protocol. Finally, slides were covered with MM24 mounting media and plastic cover slides. Microscopic images were taken using a LEICA DM4000B microscope, Olympus camera DP73, and Olympus Cellsens software.

#### 4.2.2. Immunohistochemical Staining

Immunohistochemical staining of PGP9.5 (UCHL1) was performed following the protocol optimized at WFIRM. Slides were deparaffinated and rehydrated using the Leica Autostainer XL protocol. Immunohistochemistry staining was then performed manually using the following steps. Initially, antigen retrieval was achieved using sodium citrate (0.01 M, pH = 6) at 98 °C for 15′ and then allowed to cool down at room temperature. Slides were then incubated in 0.2% Triton X-100 in PBS for 7 min to help permeabilize membranes. For the next step, endogenous peroxidase was quenched using 3% hydrogen peroxide in methanol for 30 min. Non-specific protein binding was prevented by incubating the slides in Serum-Free Protein Block solution (Dako, Santa Clara, CA, USA) for 15 min. Then slides were incubated on primary antibody overnight at 4 °C (Positive Slides: anti-PGP9.5 (UCHL1) Ab Rabbit Ig against human Abd Serotec 7863-0504 1:1000 in Dako Antibody Diluent; Negative controls: Rabbit IgG Sc-2027-1/1000 in Dako Antibody Diluent). To keep the slides moist during the incubation time, they were covered with parafilm and maintained in a humidity chamber (Sigma-Aldrich H6644). The next day, positive and negative slides were washed separately with phosphate buffer saline (PBS) for 5 min three times. Subsequently, incubation on secondary antibody was performed for 1 h at room temperature (goat anti-rabbit biotin IgG-BA1000 VECTOR (1/300) in Dako Antibody Diluent). After washing the remaining unbound antibody with PBS, the slides were incubated on Avidin-Biotin-Peroxidase solution for 30 min (Ready to Use Elite ABC Reagent by Vector Laboratories). Finally, DAB Chromagen Substrate (Vector Laboratories SK-4100) was prepared as directed by the manufacturer, then added by the drop to both positive slides and negative controls in parallel. Development was monitored under light microscopy. The optimal color was reached after 1 min and 30 s. Development was stopped by dipping the slides in water. All reagents were at room temperature unless mentioned otherwise. At this point, slides were counterstained with Gill’s hematoxylin using a Leica Autostainer XL. Finally, slides were over-slipped with MM24 mounting media. Microscopic images were taken using a LEICA DM4000B microscope, Olympus DP73 camera, and Olympus Cell sens software.

### 4.3. Cell Isolation, Culture, and Cryopreservation

Our testicular cell isolation protocol was on based on Shinohara’s group’s work [46,47,48,49] and previous works from our group [50,51] including both mechanical and enzymatic digestion of testicular tissue. For each experiment, five 3-day-old XY testes and five 3-day-old XXY testes were used, all DMSO frozen previously. Initially, cryovials were quickly thawed using running warm tap water, and testicular tissue was transferred into a petri dish with 1× MEM 8 µg/mL DNAse (Roche). Tunica albuginea was carefully removed using tweezers and a surgical blade before weighing the tissue. The next step was separating single seminiferous tubules of the testis from the surrounding conjunctive tissue with tweezers under a dissecting microscope (Leica S6D) for mechanical digestion. Once single tubules were obtained, the sample was transferred into a 15 mL centrifuge tube, and tubules were allowed to sink by gravity to the bottom. The supernatant was carefully removed, and tubules were then resuspended on enzymatic solution: 1× MEM; 12 µg/mL DNAse (Roche); Collagenase type I (CLS1) 225 U/mL; Hyaluronidase type IV from sheep (Sigma) 450 U/mL; Trypsine TRL3 (Worthington) 250 U/mL. Testicular tissue on the enzymatic solution was placed underwater in a shaking water bath at 120 rpm and 32 °C for a one-hour incubation. The solution was then centrifuged for 5 min at 16× *g* without brake, and supernatant carefully removed to discard early released cells. The remaining testicular tubules were re-suspended on a second enzymatic solution: 1× MEM 12 µg/mL DNAse (Roche); Collagenase type I (CLS1) 225 U/mL; Hyaluronidase type IV from sheep (Sigma) 450 U/mL and placed entirely under water in a shaking water bath at 120 rpm and 32 °C for enzyme incubation for 45 min. At this point, digested tubules were vigorously pipetted to help release remaining cells from the tubules and centrifuged for 5 min at 350× *g* with a brake. The supernatant was removed, and the pellet was resuspended on Trypsin 0.25% EDTA (Invitrogen) and incubated at 37 °C for 25 min to dissociate clumps of cells further. At this point, the sample was centrifuged for 5 min at 350× *g* with brake, and the supernatant was carefully removed. Cells were finally resuspended on Gonomedia (Table 1). Trypan Blue staining (1:1) and a Hematocytometer was used to assess the number of cells and viability. Cells were then seeded at 20,000–25,000 cells/cm^2^ on culture cell plastic plates (Falcon) and kept in an incubator at stable conditions of 37 °C 5% CO_2_.

Culture media was refreshed every three to four days. When confluency of the attached cells was approached, 80% of cells were passaged and split using Trypsin 0.25% EDTA (Life Technologies). A surplus of cells were cryopreserved in MEM 20% FBS and 8% DMSO and kept overnight in Mr. Frosty at −80 °C. The next day, cryotubes were transferred into liquid nitrogen tanks for long-term storage.

### 4.4. Quantitative Reverse Transcriptase Polymerase Chain Reaction (q RT-PCR)

RNA was extracted using an RNEasy mini kit (Qiagen, Germantown, MD, USA) from Snap Frozen tissue or cells. The quality and quantity of the resultant product were tested with a spectrophotometer (Nanodrop 2000, ThermoFisher, Waltham, MA, USA). RNA was then converted to cDNA using a Reverse Transcriptase Kit (Life Technologies, Carlsbad, CA, USA) and through the following thermocycler (Simpli amp thermal cycler, life technologies) conditions: 25 °C for 10 min, 37 °C for 120 min, 85 °C for 5 min and then hold on 4 °C. The resulting cDNA samples underwent PCR amplification using Taqman primers (Supplementary Table S1) and an applied Biosystems 7300 Real-Time PCR system. The cycling conditions followed were 95 °C for 10 min, then 40 cycles of 95 °C for 15 s and 60 °C for 1 min. All primers (Supplementary Table S1) were previously tested for not amplifying genomic DNA. POLR2A was selected as a housekeeping gene [68] and the expression of genes was normalized to this gene; relative expression was determined with the Delta CT method.

Amplified cDNA from RT qPCR was subsequently used for an electrophoresis study on the gel to visualize the specific product bands. A 2% agarose gel was used, given that our target primers were between 50 and 120 base pairs of length (Supplementary Table S1). Ethidium Bromide was included in the gel formulation at a concentration of 5 µL/100 mL. After samples and DNA ladder were loaded, a 140 V Voltage (Enduro power supply, Labnet, Edison, NJ, USA) was applied for 20 min when the DNA dye reached 2/3 of the total gel size. Images of the gel were taken using a UV light Camera system (Gel logic 200 imaging system).

### 4.5. Digital Reverse Transcriptase Polymerase Chain Reaction (d RT-PCR)

A QuantStudio 3D Digital PCR system (Life Technologies, Carlsbad, CA, USA) was used to estimate the population of cells expressing specific markers for each cell type expected in culture. Every chip was loaded with 2 Taqman assay primers: one with FAM signal for the specific targeted gene and one with VIC signal for the housekeeping gene POLR2A. The chip load was completed following the manufacturer’s manual with commercially available dPCR Master Mix and cDNA from RNA extracted from Snap Frozen cells. The quantity of cDNA loaded in each 20,000 wells chip was 50 ng. Assuming each mammalian cells has around 10–30 pg of total RNA, each well should represent at most one cell with a significant number of empty wells as a safety margin. Cycling conditions were 95 °C for 10 min, then 40 cycles of 95 °C for 15 s, and 60 °C for 1 min.

### 4.6. Flow Cytometry Analyses

The population of putative spermatogonial stem cells (SSCs) in the cells in culture was estimated as the MHC I/CD9+/CD49f+ population [54,55,56,57,58,59] using a BD Accuri C6 Flow cytometry system without sorting. BD antibodies were used (Supplementary Table S2) at a concentration of 5 µL of antibody per 50,000 cells in 100 µL. Cells were incubated with the antibody for one hour at room temperature and then washed with FACS Buffer (1% FBS in PBS). The obtained data were analyzed using BD Accuri software. Unstained cells and isotype controls (Supplementary Table S2) were used to optimize channel compensation and as a negative control. In every condition, at least 10,000 events were evaluated.

In separate experiments, the same staining method was used to perform Fluorescence-Activated Cell Sorting (FACS) using BD FACS ARIA. Cells expressing MHC I-/CD9+/CD49f+ were sorted and used for DNA FISH analysis.

### 4.7. X and Y Chromosome Fluorescent In Situ Hybridization (FISH)

After cells in culture were harvested by trypsin, 50,000 cells were attached to the glass microscopic slides by cytocentrifuge (Cytospin) for 10 min at 1000 RPM on glass poly-l-lysine slides (citopro, ELI Tech Biomedical Systems, Puteaux, France) using the cytopro 7620 cytocentrifuge system (Wescor). The slides were left to dry at room temperature overnight. Then, slides were soaked in 2× saline sodium citrate (made from stock 20xSSC from ABBOTT/VYSIS Company, Chicago, IL, USA) at 37 °C for 35 min. Subsequently, cells were incubated in Pepsin (AVANTOR PERFORMANCE MATERIALS, INC 2629, company) 0.5 mg/mL solution of HCl 0.1 M at 37 °C for 35 min. Slides were then washed at room temperature in 1XPBS for 5 min, and Post-Fixation Solution (0.9% formaldehyde *w/v*; 4.5 mg/mL MgCl_2_ in PBS) was added for 5 min of incubation at room temperature. Slides were rewashed at room temperature in PBS for 5 min, and the dehydration process was performed by submerging slides in increasing concentrations of ethanol (70%, 80%, 100%) for 1 min each at room temperature. At this point, working solutions of X and Y chromosome probes (EMPIRE GENOMICS Company kit MCEN-Y-10-GR and MCEN-X-10-OR 1:1:4 probe buffer dilution) were added to the sample, and slides were kept at 75 °C for 5 min overnight incubation at 40 °C (16 h minimum). The following day, slides were washed in 0.3% IGEPAL/solution (SCI-GENE, Stanford, CA, USA) on 0.4X saline sodium citrate/for 2 min at 73 °C and 0.1% IGEPAL (SCI-GENE, Stanford, CA, USA) solution on 2X saline sodium citrate for 1 min at room temperature. Slides were finally mounted with ABBOTT/VYSIS DAPI II and coverslips applied. Imaging of the slides was performed using a Zeiss Axiophot microscope and Applied Spectral Imaging Software.

## 5. Conclusions

To the best of our knowledge, this is the first report of long-term culture and propagation of mouse XXY testicular cells, well beyond the previously noted and expected time of germ cell loss. Moreover, the comparable in vitro growth rates of XXY testicular cells and cultured cells from wild-type mice testes open the door to pursue new cell-based therapies to treat infertility in Klinefelter syndrome patients. The growing mosaicism observed in the cells in culture may lead to a better understanding of the stem cell selection dynamics in the Klinefelter patient’s testis. Finally, we hope this body of work may be reproduced in human Klinefelter tissue.

## Figures and Tables

**Table 1:** Gonomedia formulation.

Reagent	Company	Catalog #	Final Concentration
**Stem Pro-34 SFM**	Invitrogen	10639-011	Base Medium
**Stem Pro Supplement**	Invitrogen	10639-011	26 µL/mL
**Bovine Albumin**	Roche	10735094001	5 mg/mL
**D(+) Glucose**	Sigma	G7021	6 mg/mL
**Ascorbic acid**	Sigma	A4544	1 × 10^−4^ M
**Transferrin**	Sigma	T1147	100 µg/mL
**Pyruvic acid**	Sigma	P2256	30 mg/mL
**d-Biotin**	Sigma	B4501	10 µg/mL
**2-beta Mercatoethanol**	Sigma	M7522	5 × 10^−5^ M
**DL-lactic acid**	Sigma	L4263	1 µL/mL
**MEM-non essential aa**	Invitrogen	11140-035	10 µL/mL
**Insulin**	Sigma	I1882	25 µg/mL
**Sodium Selenite**	Sigma	S1382	30 nM
**Putrescine**	Sigma	P7505	60 µM
**L-Glutamine**	Invitrogen	25030-024	2 mM
**MEM Vitamine solution**	Invitrogen	11120-037	10 µL/mL
**b-Estradiol**	Sigma	E2758	30 ng/mL
**Progesterone**	Sigma	P8783	60 ng/mL
**Human EGF**	Sigma	E9644	20 ng/mL
**Human bFGF**	Sigma	F0291	10 ng/mL
**Human LIF**	Chemicon	LIF1010	10 ng/mL
**GDNF**	Sigma	G1777	10 ng/mL
**FCS**	Invitrogen	10106-169	20%
**Pen/Strep**	Invitrogen	15140122	0.5%
**Platelet-Derived Growth Factor (PDGF)**	Sigma	SRP3228-10UG	10 ng/mL
**Follistatin (Ft)**	Sigma	F1175-25UG	100 ng/mL
**Follicle Stimulant Hormone (FSH)**	Sigma	F8174-1VL	200 ng/mL

## Data Availability

The data presented in this study are available on request from corresponding author.

## Supplementary Materials

The following are available online at https://www.mdpi.com/article/10.3390/ijms23010173/s1.
