# Supplementary material for: In Vitro Propagation of XXY Undifferentiated Mouse Spermatogonia: Model for Fertility Preservation in Klinefelter Syndrome Patients
Source: Int J Mol Sci. 2021 Dec 24;23(1):173. doi: 10.3390/ijms23010173 (PMC8745151; doi:10.3390/ijms23010173)
Supplement: Supplementary file 1 [file ijms-23-00173-s001.zip › ijms-1444856-supplementary.pdf]

Supplementary Materials:

Table S1. Log of Taqman primers used for PCR analysis in the study.

Taqman Primers	Catalog #	Lot #	Length (base pairs)
STRA8-FAM	Mm00486473_m1	P151128-001B04	68
UCHL1-FAM	Mm00495900_m1	P160426-006A10	78
ZBTB16-FAM	Mm01176868_m1	P150501-008 A10	63
THY1-FAM	Mm00493681_m1	P160426-006 H06	68
CD9-FAM	Mm00514275_g1	P160812-007 H05	64
ITGA6-FAM	Mm00434375_m1	P160798-011 F01	88
ITGB1-FAM	Mm01253230_m1	P151125-007 A04	75
GATA4-FAM	Mm00484689_m1	P150717-009 B06	84
SOX9-FAM	Mm00448840_m1	P160719-007 A05	101
CYP19a1-FAM	Mm00484049_m1	P160527-000 G10	74
CYP11A1-FAM	Mm00490735_m1	P160809-007 A05	82
CD34-FAM	Mm00519283_m1	P160527-002 A05	61
ACTA2-FAM	Mm01546133_m1	P151120-005 B11	88
POLR2A-FAM	Mm00839493_m1	P150306-005 A03	85
POLR2A-VIC	Mm00839502_m1	P161025-099 H09	70

Table S2. Log of Antibodies used for Immunohistochemistry and FACS in the study.

Antibody (dilution)	Catalog #	Company
Anti-Mouse MHC I (H-2Db) FITC (1:20)	#11-5999	eBiosience
Mouse IgG2a K Isotype FITC (1:20)	#11-4724	eBiosience
Anti-Mouse CD9 PE (1:20)	#12-0091	eBiosience
Rat IgG2a K Isotype PE (1:20)	#12-4321	eBiosience
Anti-Human/Mouse CD49f APC (1:20)	#17-0495	eBiosience
Rat IgG2a K Isotype APC (1:20)	#17-4321	eBiosience
Anti-Human/Mouse _PGP9.5 (UCHL1) (1:1000)	#7863-050	Serotec
